# Prevalence of Hepatitis B, C, HIV and syphilis markers among refugees in Bari, Italy

**DOI:** 10.1186/1471-2334-10-213

**Published:** 2010-07-20

**Authors:** Silvio Tafuri, Rosa Prato, Domenico Martinelli, Livio Melpignano, Maria De Palma, Michele Quarto, Cinzia Germinario

**Affiliations:** 1 Department of Biomedical Sciences, Hygiene Section, University of Bari Aldo Moro, Puglia Regional Observatory for Epidemiology, Bari, Italy. Piazza Giulio Cesare 13-15, 70124 Bari, Italy; 2 Department of Medical Sciences, Hygiene Section, University of Foggia, Puglia Regional Observatory for Epidemiology, Foggia, Italy. Viale L. Pinto Ospedali Riuniti di Foggia, 71100 Foggia, Italy

## Abstract

**Background:**

The aim of this study was to assess the prevalence of Human Immunodeficiency Virus (HIV), Hepatitis B Virus (HBV) and Hepatitis C Virus (HCV) serological markers and the prevalence of VDRL positive subjects in a population of refugees of various nationalities, living in the Asylum Seeker Centre in Bari Palese, Southern Italy.

**Methods:**

The study was carried out in the period May-July 2008 and recruited only voluntarily enrolled healthy refugees. HBsAg, anti-HBc, anti-HCV and anti-HIV virus antibodies were detected. VDRL syphilis screening was also carried out on the serum samples.

**Results:**

A total of 529 refugees, 442 males and 87 females, aged between 7 and 52 years, were studied. Of these, 510 were from Africa and 19 from Asia.

Forty-four individuals (8.3%) were HBsAg positive and 241 (45.6%) were anti-HBc positive. A total of 24 (4.5%) individuals were anti-HCV positive. Eight asylum seekers (1.5%) were HIV positive. VDRL tests were performed on 269 subjects and 4 (1.5%) were positive. 12.3% of the study population had serological markers of chronic and transmissible infections with potential blood-borne or sexual transmission.

**Conclusions:**

In Italy, a suitable protocol is necessary for the early diagnosis of infectious diseases on entering Asylum Centres, so allowing the adoption of prevention measures to safeguard the health of the individuals, the residents and workers in the Centres and the general population.

## Background

There are estimated to be about 3.8 million immigrants in Italy, around 6% of the resident population. In 2008 alone the non-Italian population increased by about 500.000. Reliable estimates of immigration movements can be ascertained from the figures for hirings of non-Italians by companies and families in recent years. These are 251,000 in 2005, 520,000 in 2006 and 741,000 in 2007. The immigration movements reported in the last ten years are among the highest in Italian history [1].

Puglia is a region in southeastern Italy bordering the Adriatic Sea in the east, the Ionian Sea to the southeast, and the Strait of Otranto and Gulf of Taranto in the south. Because of its geographic position, Puglia has since 1991 been subject to immigration influx, at times on a large scale, and is recognized to be a "Border Region" [2].

In recent years there has also been an increase in the number of people asking for asylum and refugee status in Italy.

Under Italian law, asylum seekers are housed in Asylum Seeker Centres. There were forty-four centres in 2008, with almost 8000 places available [3]. On arrival at the Centre, medical controls are carried out only for scabies and dermatophytosis that are mandatory in Italy. No screening takes place for infectious diseases of sexual and parenteral transmission, though this is recommended in the protocols of the Centers for Disease Control and Prevention (CDC) [4,5].

Refugee populations are more at risk of having HBV, HCV, HIV and sexually transmitted infections (STI). The contributing factors include: origin from countries that are highly endemic for these infections [6,7]; lack of information on STI prevention directed to the migrant communities in the host country [8]; the predominance of younger and more sexually active persons [8]; the breaking up of couples and other family ties, and the exclusion from normal society, exacerbated by the barriers of language, culture and socioeconomic conditions. The risk of these and other infections is aggravated by the harsh or chaotic living conditions many refugees experience before emigration [9].

Currently, Italy is among the countries with the lowest level of endemic hepatitis B, chronic carriers are around 1.5%, with an acute symptomatic infection rate of 1-2 per 100,000 inhabitants/year and almost no infection in childhood. Up to the middle 1980's, HBV was widespread in Italy but since then there has been a rapid decline in the acute infection rate and also in chronic infection [10].

The data for hepatitis C show anti-HCV positive subjects at 3% of people under 50 years old, but rising with age, with highspots of over 40% among the over-60s in some parts of Italy [10].

In Italy, the rate of new cases of HIV reached a peak in 1987, falling off to 1998 and then stabilizing at a rate of about 6 new cases per 100,000 inhabitants/year. This trend is similar for both males and females but the proportion of females has increased over the years [11].

Assessing the prevalence of viral hepatitis among refugees is necessary for the planning of health control measures in primary and secondary prevention. Prevention is of great importance for public health, as chronic viral hepatitis carries long-term risk of cirrhosis and remains the main cause for the development of hepatocellular carcinoma in Europe and worldwide.

The disease Syphilis has been associated with migration and population mobility through much of recorded history and today its modern epidemiology can also represent a demographic indicator for sexually transmitted infections and the international movement of disease [12].

The aim of this study was to assess the prevalence of HIV, hepatitis B and C and treponemal infection serological markers in a population of refugees of various nationalities, living in the Asylum Seeker Centre in Bari Palese in Puglia, Southern Italy. This screening was necessary to allow the adoption of both the treatment of the infected individuals and to put in place the measures necessary to safeguard the residents and workers in the Centre.

## Methods

In the year 2008, the Asylum Seeker Centre in Bari received 2,550 immigrants of which 2,222 were males (87.1%) and 328 females (12.9%), with an average age of 24 years. On arrival at the Centre each new resident was examined by the Centre's medical staff. Immigrants with symptoms or with a positive disease history were treated as necessary. During the period May-July 2008, immigrants who were in apparent good health and did not report signs or symptoms in the recent or remote past were asked if they wished to undergo the testing after its importance had been explained by the Centre's medical staff and cultural mediators. Adhesion was completely voluntary and signed informed consent, which was written in the immigrants mother tongue, was obtained from 529 volunteers of 744 refugees admitted to the Centre beetween May-July 2008. Test results were communicated to refugees and positive cases were treated in the Infectious Disease Unit of the Local Hospital. Data were stored according to the Italian laws on privacy.

The Regional Government Authority (*Assessorato alle Politiche della Salute*) gave the approval to carry out the investigation by offering the tests to immigrants and to use the results of the tests anonymously for scientific aims. Ethical approval was not required for this study because the project involved data routinely collected. The research was carried out in compliance with the Helsinki Declaration.

The hepatitis B virus surface antigen (HBsAg), the hepatitis B virus core antibody (anti-HBc) and the hepatitis C virus antibody (anti-HCV) were detected by a third-generation enzyme-linked immunosorbent assay. This immunoassay method was carried out according to the instructions of the manufacturer (Abbott prism Architect AxSYM and Architect Test, Abbott Diagnostic Division, Italy).

Antibodies to HIV (anti-HIV) were determined by chemiluminiscent microparticle immunoassay  (HIV Ag/Ab Combo, Abbott Diagnostic Division, Sligo, Ireland) and positive results were confirmed by line immunoassay (INNO-LIA HIV I/II, Immunogenetics GmbH, Germany).

Venereal Disease Research Laboratory (VDRL) syphilis screening was also carried out on 269 of the 529 serum samples (Alfa Wasserman Diagnostic, Milano, Italia).

All patients found positive were treated in the Local Health Unit following specific care protocols.

Demographic data from the Asylum Centre database and the laboratory exam results were analyzed with the statistical software Epi-Info 6.00. The Chi-square test was used for the analysis of the difference between proportions with a value of p < 0.05 considered significant.

## Results

A total of 529 refugees (71.1% of the 744 admitted to the Asylum Centre between May-July 2008), of which 442 males and 87 females, aged between 7 and 52 years (average = 23.9; SD = 6.7 years), were studied. Table 1 shows study population by sex and by geographic continent.

**Table 1:** Study population per sex and continent of origin (No. = 529)

	Female		Male		Total
	
Continent	No.	%	No.	%	No.
Africa	87	17	423	83	510

Asia	--	--	19	100.0	19

Total	87	16.4	442	83.6	529

Of the total of 529 refugees under study, 44 individuals (8.3%) were HBsAg positive and 241 (45.6%) anti-HBc positive. The prevalence of HBsAg positive subjects was significantly higher in males (9.7%; 95% CI = 7.2-13.0) than in females (1.1%; 95% CI = 0-6.2; p = 0.008). The proportion of anti-HBc positive individuals was also higher in males (48.4%; 95% CI = 43.7-53.2) than in females (31%; 95% CI = 21.5- 41.9; p = 0.002). There was no significant average age difference between sero-positive and sero-negative HBsAg, being respectively 23.8 years (SD = 7.0) and 24 years (SD = 6.7; p >.05), while for anti-HBc positive and negative individuals average age was respectively 24.5 years (SD = 7.7) and 23.6 years (SD = 5.7; p > .05). Table 2 shows the distribution of hepatitis B serological markers by geographical origin.

**Table 2:** Distribution of hepatitis B and hepatitis C markers per sex and continent of origin (No. = 529)

	Male	Female	*p*	Africa	Asia	*p*
**No.**	442 (83.6%)	87 (16.4%)		510 (96.4%)	19 (3.6%)	

**HBsAg(+)**	43 (9.7%)	1 (1.1%)	*0.008*	44 (8.6%)	0	*>.05*

**Anti-HBc (+)**	214 (48.4%)	27 (31%)	*0.002*	234 (45.9%)	7 (36.8%)	*>.05*

**HBsAg (-), anti-HBc(-)**	228 (51.6%)	60 (68.9%)	*0.002*	276 (54.1%)	12 (63.2%)	*>.05*

**Anti-HCV(+)**	23 (5.2%)	1 (1.1%)	*>0.5*	22 (4.3%)	2 (10.5%)	*>.05*

**Any infection**	63 (14.3%)	2 (2.3%)	*0.0001*	54 (10.5%)	2 (12.4%)	*>.05*

A total of 24 (4.5%) individuals, 23 males (5.2%; 95% CI = 3.4-7.9) and 1 female (1.1%; 95% CI = 0-6.3) were anti-HCV positive. In detail, the figures were 4.3% (95% CI = 2.8-6.6) for African refugees and 10.5% (95% CI = 1.3- 33.1; p >.05) for Asians (Table 2).

The average age of anti-HCV positive subjects (26.5, SD = 7.8) was higher than that of anti-HCV-negative subjects (23.8, SD = 6.5; p = 0.04).

Three males from Africa were found to be HBsAg/Anti-HCV positive.

Eight refugees (1.5%), 6 males (1.4%; 95% CI = 0.6-3.1) and 2 females (2.2%; 95% CI = 0.3-8.5), all from Africa, were HIV positive. The average age of HIV-positive individuals was 25.4 (SD = 9.3) without any statistically significant difference to HIV-negatives (average age = 23.9, SD = 6.6; p >.05).

Two African male refugees were HIV/Anti-HCV positive.

Four individuals from Africa of the 269 tested (1.5%) were positive to the VDRL test. Their average age was 26.7 years (SD = 6.2), not significantly different to VDRL-negatives (average age = 24.9, SD = 7.3; p > .05).

The subjects found with any infection were 65 (12.3%), with a higher proportion in males (14.3%; 95% CI = 10.9-17.5) than in females (2.3%; 95% CI = -0.8-5.4; p = 0.0001). Their average age was 24.5 years (SD = 7.0), not significantly different to subjects without infection (23.9; SD = 6.7; p >.05). There was no statistical difference in continent of origin with an infection rate of 12.4% in Africans (95% CI = 9.7-15.6) and of 10.5% in Asians (95% CI = 1.3-33.1; p >.05; Table 2).

## Discussion

In our study on 529 refugees, almost half of them had serological markers of past or active infections, that is 8.3% were HBsAg positive, 45.6% were anti-HBc positive, 4.3% anti-HCV positive and 1.5% HIV-positive.

Various other similar studies have been carried out in recent years. In a study performed in Verona (Italy) in 2008 among 182 Sub Sahara refugees, HBsAg positives were 9.3% and HBs/anti-HBc positives were 35.2%, with anti-HCV positives at 2.7%. In this study only illegal immigrants attending a volunteer health center have been analyzed [13].

In a study performed in Greece published in 2008 by Vasilopoulou et al, HBsAg positives were 27.3% for Asian refugees, while anti-HBc positives were 45% for Asian and 25% for African immigrants. The African refugees also had a rate of anti-HCV positives of 12.5% [14].

Palumbo et al, in a study carried out in the Italian province of Foggia in 2007 on 469 refugees, documented a HBsAg positivity rate of 11.1%, without any difference between African and Asian subjects. Also this study used a convenience sample [15].

Our results, in a larger population than the above three studies, produced similar infection rates though there are various factors in our study that can give rise to a selection bias. Many immigrants may not have volunteered because they refuse contact with authority figures because of fear of expulsion, others can have specific cultural or religious beliefs concerning medical practices. Moreover, some of them could perceive the hepatitis screening as related with STIs or with sexual activity in general, or useless for their health. The reputation also could act as a barrier [16].

The study population contained very few Asians.

Finally it was not possible to check vaccination history because the great majority of primary refugees lacked documentation for recommended immunizations.

Furthermore, two of the performed tests (anti-HCV and VDRL) lack specificity for diagnosis true active infections. In our survey screening tests and not diagnostic tests for infections were performed. Screening tests do not mean the prevalence of infection but the prevalence of the markers that is important by an epidemiological perspective.

In the immigrants studied, HBsAg positivity is higher than in the autochthonous population. A constant seroepidemiological surveillance would seem to be essential to monitor the prevalence of hepatitis and other infections so allowing their transmission to be controlled through counselling and vaccination programs wherever possible. Already, following the results of this study, hepatitis-B vaccination is offered free to susceptible refugees resident in the Asylum Seeker Centre in Bari Palese.

While the lack of a vaccine makes counselling indispensable for HCV-positive subjects and their cohabitants, this counselling would also be helpful against other parenterally or sexually transmitted infections. Even if international guidelines to date recommend only universal HBV and HIV screening, and HCV screening according to symptoms, risk factors, etc., recent evidence seems to support the effectiveness of screening for HCV in populations at risk [17,18]. Our study can add a contribution to the debate about pros and cons of HCV screening.

The percentage of HIV positives (1.5%) was higher than that found in the general Italian population [11]. Immigrants continue therefore to be a risk population for HIV infection. Although the incidence of new diagnosis of HIV infection has decreased in recent years both in the Italian and the non-Italian population living in Italy, the difference in incidence between the two populations has risen, probably because of an increase in immigration from endemic zones in Africa, where HIV has been increasing [19,20].

The percentage of those positive for the VDRL test (1,5%) was much higher than in the general Italian population, where the prevalence of syphilis is estimated to be 86 × 100,000 residents [21]. However VDRL may be positive in non-syphilis treponemal diseases and indeed in many other conditions and so show a high rate of false positives [22].

Some limitations affected our observations, such as the convenience sampling and some of the diagnostic tests lacking of specificity. Although these limitations, the results of our investigation appear important to show this situation in Italy.

## Conclusions

Our survey has been carried out during a 3-month period in a Centre with 2550 immigrants over the course of a year. Although the conclusions that can be drawn about the reported subpopulations could be partially limited, a significant proportion (12.3%) of asymptomatic refugees presented with at least one condition potentially associated with long-term complications and risk of secondary transmission.

In Italy, there is the need for the adoption of a suitable protocol for the early diagnosis of infectious diseases upon entering the Asylum Centre, to allow the adoption of prevention measures to safeguard the health of the individuals, the residents and workers in the Centres. Testing and improving knowledge of serologic status of refugees substantially help the managing of infectious hazard in crowding and at strictly contacts immigration settings.

## Abbreviations

STI: sexual transmitted infection; HBsAg: hepatitis B virus surface antigen; anti-HBc: hepatitis B virus core antibody; anti-HCV: hepatitis C virus antibody; VDRL: Venereal Disease Research Laboratory

## Competing interests

The authors declare that they have no competing interests.

## Authors' contributions

ST and RP conceived of the study, participated in its design, performed analysis and interpretation of data and drafted the manuscript. DM contributed to analysis and interpretation of data. MDP and LM have made substantial contributions to conception of the study and data acquisition. CG and MQ conceived of the study and critically revised the manuscript for important intellectual content. All authors have given final approval of the version to be published.

## Pre-publication history

The pre-publication history for this paper can be accessed here:

http://www.biomedcentral.com/1471-2334/10/213/prepub
